# Middle-Term Evolution of Efficiency in Permeable Pavements: A Real Case Study in a Mediterranean climate

**DOI:** 10.3390/ijerph17217774

**Published:** 2020-10-23

**Authors:** M. I. Rodríguez-Rojas, F. Huertas-Fernández, B. Moreno, G. Martínez

**Affiliations:** 1 Department of Urban and Regional Planning, Higher School of Civil Engineering, University of Granada, 18071 Granada, Spain; franhuf@ugr.es; 2 Department of Construction and Engineering Projects, Higher School of Civil Engineering, University of Granada, 18071 Granada, Spain; bgmoreno@ugr.es (B.M.); gmmontes@ugr.es (G.M.)

**Keywords:** permeable pavement, Sustainable Drainage Systems, efficiency, soil sealing, saturation, Mediterranean area

## Abstract

Sustainable Drainage Systems (SuDS) are solutions used to reduce the effects of soil sealing and to contribute to sustainable storm water management. In recent years, many projects have been implemented in Europe, the United States, and Australia, but most of them have either not been monitored at all or have only been monitored in the short-term, so there is little information on the evolution of efficiency and clogging. Experiences in the Mediterranean are even rarer, so the main purpose of this research is to provide information about the long-term behavior of one kind of SuDS, the permeable pavements, in the middle-term under Mediterranean climatic conditions. This work shows the results of a real project developed in southern Spain, which has been monitored for five years. The evolution of efficiency in permeable pavements and their relationship with saturation are analyzed and discussed in this research. These results will help to manage and maintain permeable pavements in areas with a Mediterranean climatology.

## 1. Introduction

The fast growth of cities and the need to facilitate increasingly intense road traffic has generated a model of urbanization based on impervious surfaces [1]. This has resulted in 67% of the 1000 km^2^ of surface area that is urbanized per year in Europe [2] being impermeable [3]. This process of ‘soil sealing’ is having serious environmental consequences on the territory and the city [4,5]: increase in temperature or ‘heat island effect’ [6], contamination of receiving waters [7,8], and an increase in the intensity and periodicity of floods [9]. Conventional drainage systems are particularly affected by this waterproofing process as they have not been designed for an increasing volume of runoff that is expected to continue to increase due to climate change [10,11].

Sustainable Drainage Systems (SuDS) have proved to be a useful tool for mitigating the impact of imperviousness on storm water runoff in urban areas [12,13], mimicking the pre-development hydrologic conditions by facilitating storage, infiltration, and evapotranspiration processes [14,15]. SuDS contributes to the mitigation of urban flooding and water pollution [7], providing a nonconventional water resource [3], amenity, wildlife, carbon sequestration and storage, urban cooling, human-health, and well-being [16,17]. Types of SuDS include green roofs, permeable surfaces, wetlands, detention and infiltration basins, and filter drains, among others [18]. Scientific literature uses different terms for these systems, such as Sustainable Drainage Systems (SuDS), Low Impact Development (LID), Water Sensitive Urban Design (WSUD), Best Management Practices (BMP), and innovative storm water management [19,20]. These systems are being used as support for the transition to more sustainable and resilient environments [21,22]. In spite of all the benefits of SuDS, its implementation is progressing slowly [23,24]. The lack of monitored projects is an important barrier [25,26]. Local governments and companies that are in charge of managing and maintaining SuDS require more information about their operation and maintenance requirements to invest in them [27]. The recent start-up of many SuDS projects, and the lack of monitoring of their performance during the operational phase, results in a lack of data on the evolution of their efficiency over time. As some studies [28,29] indicate, more information is needed to optimize the operation of these systems, identifying the ‘end of life’ and when maintenance may be necessary to restore permeability and hydrological functionality.

Most of the SuDS projects that have been monitored only include the first one or two years [30,31,32]. This period, considered as the ‘short-term’, only provides results on the initial states of operation. Some studies have detected a decrease in infiltration performance over time [33,34], but there is not enough information to determine when clogging begins. Some works show evidence of obstruction after 4 years of operation [35] and 6 years [33], so, in the ‘middle-term’, clogging may appear, and maintenance operations will be required. Other papers confirm major obstructions in the ‘long-term’, starting from 10 years [29] or 12 years [36]. Different climatic conditions may be the origin of these differences, so it is not yet possible to draw definitive conclusions about the ‘end of life’ of these systems. Very few permeable pavement projects have been developed in the Mediterranean region [3,27], where the rainfall regime is very heterogeneous (droughts and very intense rain episodes). In addition, in these cases, middle-term performance data has not been studied.

Thus, the main objective of this research is to analyze the behavior of permeable pavements in the middle-term under Mediterranean climatic conditions. This work contributes to improving the knowledge of the performance of these solutions in these areas, which have not yet been studied, since most of the studies have been carried out in climates with a more regular distribution of annual rainfall. This project, developed in southern Spain, has been in operation since 2014. In the first year of operation, comparative results were obtained on the hydrological variables and the efficiency of three types of permeable pavements [37]. In the second phase, research focused on middle-term performance. This work shows the results of the possible effects of clogging on these systems and their relation to soil saturation.

## 2. Materials and Methods

### 2.1. Site Description

The case study is located on the Cartuja university campus in the city of Granada (37°11′28.06″ N; 3°35′50.23″ W), in the south of Spain. This experience consists of a car parking area of approximately 2500 m^2^ in total, with 3 types of permeable pavements that take up 813 m^2^ (32.52%). The pavements built are ‘block pavement’ (P1), ‘concrete grid’ (P2), and ‘plastic grid’ (P3) (Table 1). The blocks are pieces of 20 × 10 × 8 cm with a porosity of 10% and 1 cm separation between them to ensure permeability. The concrete grids are panels of 60 × 40 × 10 cm with a porosity of 12% and holes filled with topsoil. Finally, the plastic grids are structures of 26 × 47 × 5 cm with a porosity of 90% and are the most permeable system of all. The Curve Number (CN) values of these pavements (the amount of runoff generated by a soil [38]) have been calculated experimentally. These pavements have been selected because of their high applicability in cities. 

**Figure 1:**
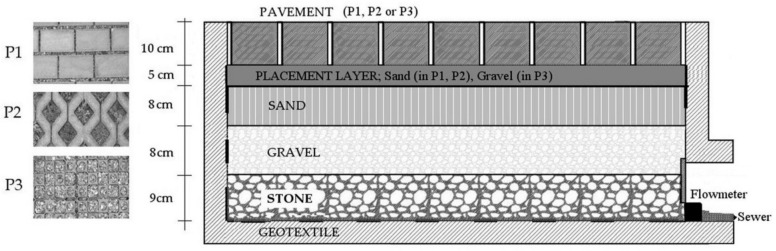
Profile of sub-base used for test areas.

Only one sub-base has been used to compare the behavior of each pavement in rain events of different intensities. The sub-base is an artificial material composed of sand with a diameter from 4 to 8 mm, gravel from 25 to 35 mm, and stone from 35 to 50 mm (Figure 1). This material has an average water absorption capacity of lower than 2%, ensuring permeability. Between the pavement and the sub-base, a 5 cm layer of sand (in P1 and P2) and gravel (in P3) has been used to facilitate the placement of the pavements. An impermeable geotextile layer has been fitted under the stone layer (Figure 1) to allow all the infiltrated water to be collected and registered before being evacuated into the sewer system.

**Figure 2:**
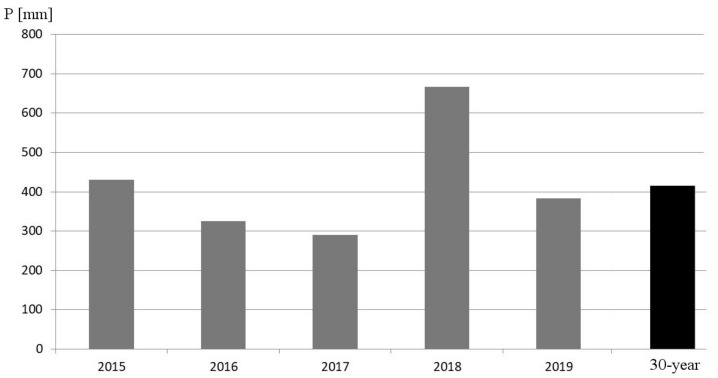
Precipitation in the last 5 years and 30-year normal precipitation [39].

In this case, there is no contributing drainage area due to the fact that the study area is located in an elevated area, and there are also perimeter barriers that prevent it from receiving external inputs. The climatic conditions are typical of a CS (climate classification) area (according to the classification by Köppen), and the average rainfall is 432 mm/year with great variability in precipitations (Figure 2). Domestic sewage and storm water runoff converge in the same sewer, and its capacity is insufficient during intense rainfall events, causing floods in the lower areas of the city.

### 2.2. Analysis of Data

The initial data has been provided by a monitoring system that consists of a pluviograph, which provides real-time data on rainfall depth, a flowmeter installed in each pavement surface, which registers drainage volume and drainage flow, and a data logger, which stores all the data on a server. The direct measurement of runoff has not been carried out due to the difficulty of carrying it out empirically on a full scale, so this variable has been calculated using the volumetric mass balance with the hydrological model SWMM (Storm Water Management Model; [40]), one of the most widely used models in the SuDS study [12,41]. It has been calibrated by making an adjustment between the modeled flows and the data provided by the monitoring system, using the parameters ‘drainage flow’, ‘Runoff’, and ‘Curve Number’. The functions that have been used for the calibration and the values obtained in the validation process are as follows:

- Least-squares. The values obtained in the three pavements are close to 0, so the adjustment of this variable is considered optimal [42].
- Nash-Sutcliffe efficiency coefficient. The medians obtained are 0.82, 0.83, and 0.72 for pavements P1, P2, and P3, respectively. According to Molnar [43], this adjustment is considered to be excellent for P1 and P2 pavements and very good for P3 pavements.
- Correlation coefficient. The medians obtained are 0.91, 0.68, and 0.78 for P1, P2, and P3 pavements, respectively. These values are above 0.6, so the adjustment is considered to be good [44].

Once the model is calibrated, the rainfall recorded in the study area from October 2014 to October 2019 has been analyzed with it, a total of 114 events. Twenty-four of them have not been considered in the study because they have not generated measurements in the flow meter. As can be seen in Table 2 and Table 3, the variability of the data analyzed has been very high, both in rainfall depth (more than 800%), in rainfall intensity (almost 50%), and in duration (almost 150%). This heterogeneity is typical of Mediterranean climatology, characterized by very dry periods that alternate with periods of torrential rains precipitating large volumes of water in very short times. This is why a specific study of the functioning of the SuDS in this climate environment is required since the behavior of these systems presents singularities when compared with other countries where the rainfall regimes are more homogeneous.

There are different ways to assess the hydrological performance of permeable pavements [45,46]. In this work, infiltration capacity has been measured by analyzing the evolution of efficiency, defined as the improvement of the performance of the permeable pavement with respect to an impermeable one, in order to quantify the improvement generated by SuDS. Thus, a global analysis of hydrological behavior has been carried out based on three hydrological variables: ‘generated volume’ (volume of water, which flows to the sewer system: ‘drainage volume’ plus ‘runoff volume’), ‘peak flow’ (maximum value for flow produced by the pavement), and ‘water residence time’ (time taken by the soil to drain the total rainfall depth). From each variable, the efficiency has been defined as the variation that occurs related to the impermeable pavement (Table 4). 

For the calculation of the efficiencies, an impermeable pavement (P0) is modeled as a reference. This pavement does not have a flow meter, so its performance has been modeled with SWMM. Values of 1 in efficiency mean that the improvement related to the impermeable pavement is 100%. The efficiencies are obtained for each of the 3 pavements analyzed, thus allowing an analysis of the evolution of their behavior and the possible clogging effects.

## 3. Results

### 3.1. Hydrologic Performance

**Figure 3:**
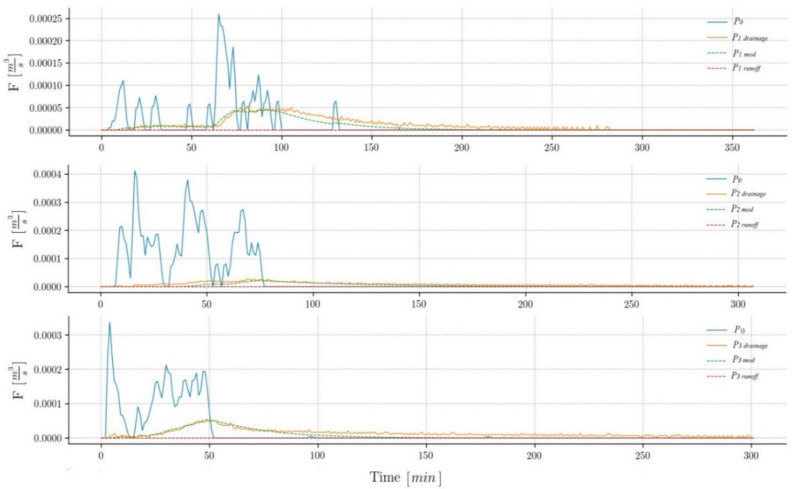
Hydrological response of the permeable pavements. P_0_ = Flow generated by P0; Pi drainage = Flow generated by Pi (measured); Pi mod = Flow generated by Pi (modeled); Pi runoff = Runoff generated by Pi (modeled).

**Figure 4:**
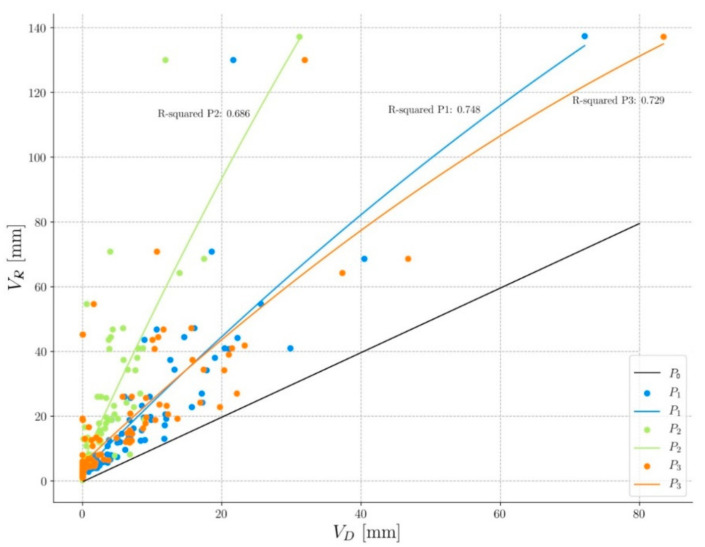
Relationship between drainage volume (V_D_) and rainfall volume (V_R_).

**Figure 5:**
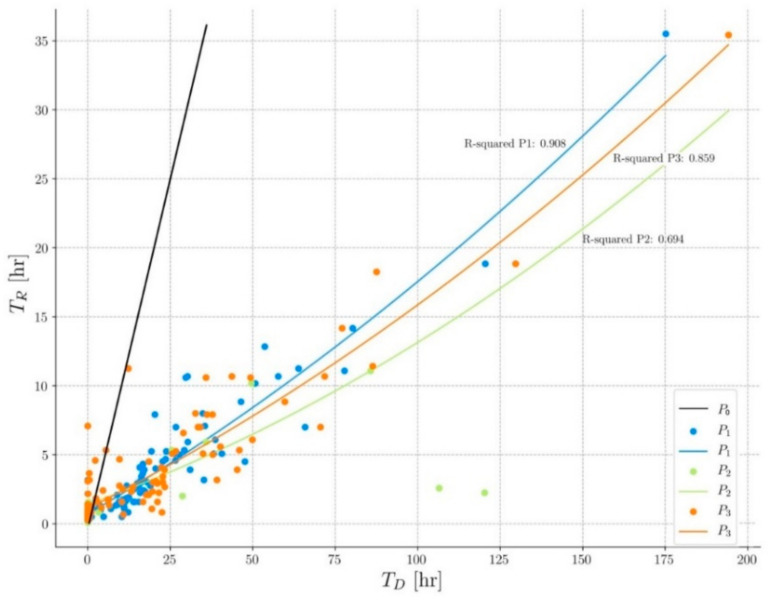
Relationship between drainage time (T_D_) and rainfall time (T_R_).

Hydrologic characteristics from measured storm events at each permeable test surface are compared to those from a simulated impervious pavement. An example of the hydrographs of each pavement is shown in Figure 3. Comparisons are based on relations to rainfall and are described below:

- **Runoff.** All events analyzed have produced runoff on P0; however, only 17% have generated runoff on P1 and 22% on P2, with values close to 1 mm, demonstrating that these pavements have been able to infiltrate almost all the precipitation. On P3, the runoff has only appeared in 3% of the events since it is a granular pavement and, therefore, has the highest water absorption and retention capacity.
  According to the historical series of precipitations in the area, in all three pavements, the runoff has not appeared in events with return-periods of less than 20 years, which indicates these systems are particularly suitable in climates, such as the Mediterranean, where the rains generate many service problems in the streets due to the water accumulation.
- **Drainage volume** (V_D_). A regression analysis has been carried out, showing that the drainage volume is clearly lower than the rainfall volume (V_R_) in all three permeable pavements (Figure 4). The difference between the two volumes corresponds to the evapotranspiration water stored in the sub-base/pavement assembly. This figure also shows that pavements P1 and P3 have very similar behavior and that pavement P2 is the one that stores the most water. The black line represents the behavior of pavement P0 that drains practically all the water it receives. As shown, all the events recorded are above this line, which indicates that permeable pavements have stored water in all the events, considerably reducing the amount of water that goes to the drainage network.
- **Drainage time (T_D_).** The increase in drainage time (compared with P0) in P1 is, on average, 394%, 429% in P2, and 366% in P3, which again indicates its great water retention capacity (Figure 3). Figure 5 shows a regression analysis where the drainage time is shown to be significantly higher than the rainfall time (T_R_) in all three permeable pavements. The values obtained are very similar among themselves, with the pavement P2 as the one with the longest drainage time.
  The broken line in Figure 5 represents the behavior of the impervious pavement that drains practically all the water at the same time as it receives it. The data points to the left of the black line indicate that the rainfall time (T_R_) has been greater than the drainage time (T_D_). This occurs because the drainage has not occurred; that is, the water has been retained in the soil. Almost all the events recorded are below this line, which indicates that the pavements continue to drain water long after the rain has stopped. This shows the buffering capacity of these pavements.
- **Peak flow**. Permeable pavements have generated lower peak flows than impermeable pavements (Figure 3). The average peak flow of P1 is 10% of P0, 4% of P2, and 7% of P3. Eighty-three percent of the events have generated a peak flow of less than 0.1 l/s in P1, less than 96% in P2, and less than 85% in P3. This shows that the hydrological behavior of permeable pavements is very stable during rain events, which has direct repercussions on the operation of the sewerage network, making it work in a much better way and significantly reducing flooding episodes.

### 3.2. Evaluation of the Efficiency Evolution

The values of flow efficiency (Ɛ_F_), volume efficiency (Ɛ_v_), and time efficiency (Ɛ_T_) of all the events are defined in Table 4. In order to analyze their evolution throughout the time of service, the results obtained for each year in each of the permeable pavements are presented. 

#### 3.2.1. Pavement P1

**Figure 6:**
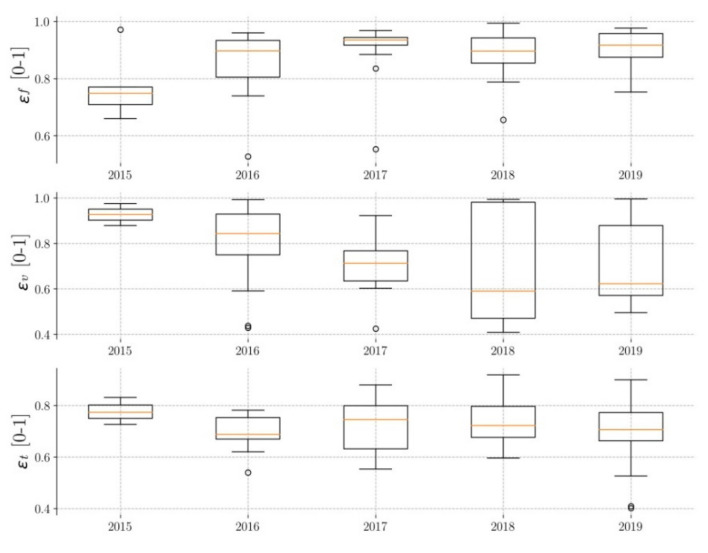
Evolution of pavement P1 efficiency.

Figure 6 shows the evolution of the efficiencies obtained for pavement P1. The median flow efficiencies (Ɛ_F_) are between 0.75 and 0.92 (22.67% variability), and the difference between the maximum and minimum values obtained is approximately 20%, indicating that the behavior of pavement P1 with respect to this variable is fairly homogeneous. No decrease or trend in average flow efficiency over time has been detected, but small variations have been seen, reaching a peak in 2017. In the case of the volume efficiencies (Ɛ_v_), the medians are between 0.59 and 0.92 (56% variability), with a difference between the maximum and minimum values obtained of up to 60%. This greater variability in the data, especially in the year 2018, shows greater heterogeneity in the response of pavement P1 to this variable. Likewise, a clear decrease in the data up to 2018 has been observed, with an increase in 2019. The time efficiencies (Ɛ_T_) show somewhat more homogeneous behavior. The medians are between 0.68 and 0.77 (13% variability), and the data variability is approximately 40%. There is no clear trend of increasing or decreasing efficiencies over time, with very little difference in data between one year and another. 

Consequently, the flow efficiencies have been the highest and those with the least data variability, and therefore the performance of pavement P1 has been better with respect to this variable. The volume efficiencies have been the lowest and with the greatest dispersion of data, as this is the variable that has provided the worst results for this pavement. On the other hand, no evident decrease in the efficiencies over time has been observed in this pavement, although small inter-annual variations have been observed. It has been noticed that flow efficiency has presented a maximum in 2017 and volume efficiency a minimum in 2018, as well as a high degree of data variability during that year. In Figure 2, the rainfall is minimal in 2017, doubling in 2018, the wettest year of the five years of operation. Thus, during the driest year, the flow efficiencies are at a maximum, and during the wettest year, the volume efficiencies are at a minimum. This fact leads us to think that soil water saturation may be the cause of both the variability of the efficiencies and the dispersion of data. 

#### 3.2.2. Pavement P2

**Figure 7:**
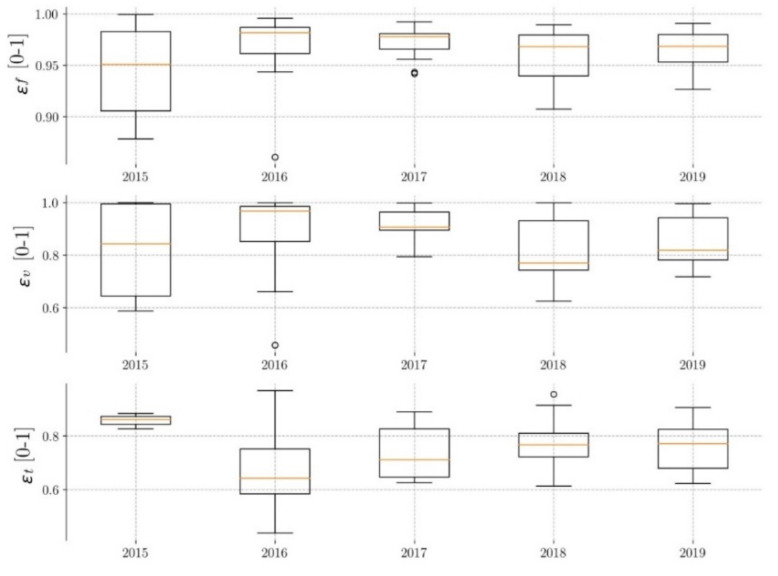
Evolution of pavement P2 efficiency.

In the case of pavement P2, Figure 7 shows that the median flow efficiencies (Ɛ_F_) are between 0.95 and 0.97 (2% variability), with a difference between the maximum and minimum values of approximately 12%. This indicates that the performance of pavement P2 against this variable is also very homogeneous, with higher efficiencies than in pavement P1 and somewhat smaller dispersion of data. As in pavement P1, pavement P2 has presented a maximum in 2017, and no decrease in efficiency has been observed over time. With regard to volume efficiencies (Ɛ_v_), the medians are between 0.78 and 0.98 (26% variability), with a difference between the maximum and minimum values of 40%. These efficiencies are greater than those of pavement P1 and present lower data dispersion, so pavement P2 behaves better against this variable than P1. Similarly, a minimum is observed in the year 2018. In the time efficiencies (Ɛ_T_), the medians are between 0.67 and 0.86 (28% variability), and the data variability is about 40%, similar to the pavement P1. There is also no clear trend of increasing or decreasing efficiencies over time.

In summary, it can be said that the values presented by pavement P2 are generally better than those of P1 (except in time efficiency), and as with P1, the flow efficiencies are the highest and the volume efficiencies the lowest. No decrease in the efficiencies over time has been observed, and the same trends are observed as for pavement P1—a maximum flow efficiency in 2017 and a minimum volume efficiency in 2018.

#### 3.2.3. Pavement P3

**Figure 8:**
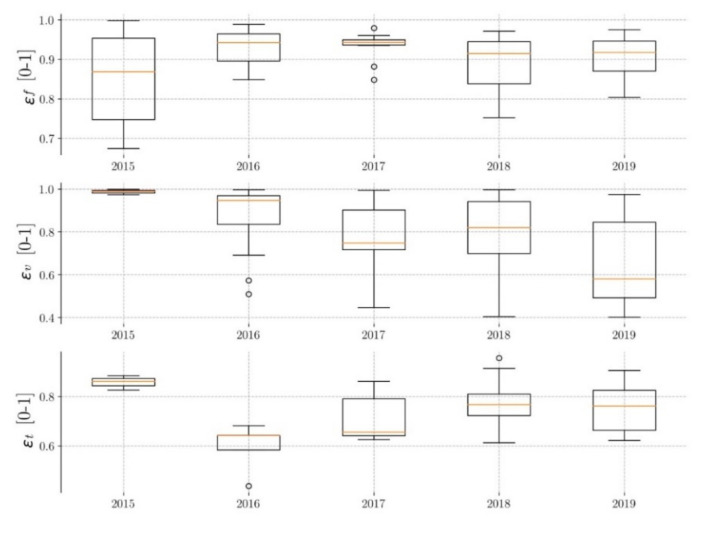
Evolution of pavement P3 efficiency.

Figure 8 shows that the median flow efficiencies (Ɛ_F_) are between 0.86 and 0.95 (10% variability), with a difference between the maximum and minimum values of approximately 35%. As with the other two pavements, a maximum is observed in the year 2017. The medians of the volume efficiencies (Ɛ_v_) are between 0.50 and 0.99 (98% variability), with a difference between the maximum and minimum values of 60%. In this case, no minimum is observed in the year 2018. The time efficiencies (Ɛ_T_) have medians between 0.65 and 0.86 (32% variability), and the data variability is approximately 35%. There is also no clear trend of decreasing efficiencies over time. However, pavement P3 shows slightly different behavior in flow efficiencies (Ɛ_F_). Pavements P1 and P2 show the expected decrease, but not pavement P3. This could be due to the difference in the granulometry of the layer located between the pavement and the sub-base. A layer of gravel (larger size) has been laid under pavement P3 (sand for P1 and P2). A specific study is needed.

In summary (Table 5), it can be said that flow efficiencies are the highest, and pavement P2 has the most regular behavior. With regard to year-on-year variations, maximum flow efficiency is observed in 2017.

### 3.3. Influence of Soil Water Saturation on Efficiency

**Figure 9:**
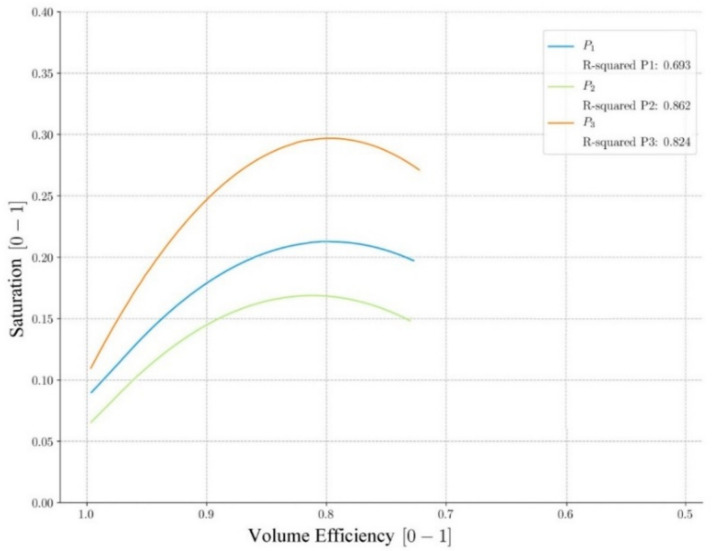
The relation between water saturation and volume efficiency.

In the analysis of the efficiencies carried out, small inter-annual variations and high dispersion of data in the volume efficiencies have been observed. Since these variations have been linked to the amount of water precipitated in different years, they could be due to the influence of soil water saturation. Therefore, a specific analysis has been carried out that compares the instantaneous volume efficiencies to the soil water saturation of each event. Direct measurement of soil saturation is not possible due to the design and exploitation requirements of the water and sanitation company. Therefore, this variable has been calculated in the hydrologic model since it has been validated with real data. Figure 9 shows the regression analysis performed:

- Pavement P2 (green) is the one that presents the lowest saturation values since it is a less porous pavement composed of sand.
- Pavement P3 (orange) is the one that presents higher saturation values since it is the most porous pavement composed of gravel.
- All three pavements achieve efficiencies above 0.65 in 95% of the events analyzed.
- All three pavements generate efficiency of around 0.8 at the moment of maximum saturation.
- The correlation results obtained have been validated with coefficients of determination R^2^ between 0.693 and 0.862.

Therefore, it can be concluded that there is indeed a correlation between efficiency and soil water saturation in the study area, which explains the inter-annual variations and the dispersion of data obtained. The fact that dispersion is maximum in volume efficiency is due to the fact that this variable depends directly on the amount of water that can be stored by the soil. If the soil has a high initial saturation, it will store less water and, therefore, its volume efficiency will be lower. However, flow efficiency presents a lower dispersion since the high values of peak flow, reached during the rain, regularize the behavior of the soil, which is less influenced by its initial saturation.

## 4. Discussion

After the analysis of the results, it can be said that the capacity of permeable pavements for mitigating rain events is enormous. These systems have been able to completely infiltrate rain events with a return period of less than 20 years, increase drainage time by up to 400%, and decrease peak flow by about 90%. These data show that these pavements are a real alternative for reducing the impact caused by the soil sealing of cities.

On the other hand, this research has demonstrated that after 5 years of operation, the blockage has not yet occurred in the permeable pavements tested, as no decrease in efficiency has been observed over time. As mentioned above, the studies carried out on the behavior of permeable pavements in the medium term do not show conclusive results. This work supports the evidence that clogging occurs at some point after 5 years of operation [29,33,36], and detects important interannual variations in soil water saturation-related efficiencies. These results could explain the difference in the obtained data by the case studies in relation to the evolution of permeable pavement performance. The effect of soil saturation on efficiency has not yet been fully investigated [47], as most work on permeable pavements focuses on the hydrological performance of the pavement [25,48,49]. Only a few studies have examined the relationship between hydrological performance and initial water saturation. Palla and Gnecco [12] and Brunetti et al. [50], for example, obtained a reduction in pavement hydraulic capacity with soil water saturation. Both works were carried out in Italy, a country with a typically Mediterranean climate. This shows that in locations with alternating wet and dry periods, the variability of initial water saturation directly affects pavement efficiency, which must be taken into account for the management and maintenance of these systems. Therefore, the study of the influence of climate on the performance of permeable pavements should be the subject of a specific study.

The main limitation of this research lies in the geometry and location of the case study. These characteristics could cause variations in pavement efficiency results if a comparison is made with pavements located elsewhere. These are the most common constraints on research into permeable pavements, as experimental studies are conducted at a specific location.

In summary, the results of this research provide information on the performance of permeable pavements in Mediterranean environments, which is necessary for the application and management of these systems in Mediterranean areas where the number of experiments is still low.

## 5. Conclusions

The ongoing process of ‘soil sealing’ is having serious environmental consequences in our cities, and the use of SuDS as a tool for its mitigation and compensation has become popular in recent years. For this reason, there are a large number of studies that analyze the behavior of these systems, such as permeable pavements, which have been installed in many cities around the world in the last 25 years. There are numerous pieces of work that have analyzed the efficiency of these pavements when compared to impermeable pavements. However, given the recent implementation of these systems and the impossibility of monitoring them for more than one year, not many studies have analyzed the evolution of the behavior of these pavements over time and the effect of clogging, even less so in the Mediterranean region. 

This research contributes to improving the knowledge about this phenomenon in permeable pavements, showing the results obtained in a real case in a Mediterranean area. The results obtained show that in the pavements tested, no clogging occurs in the middle-term, as some studies indicate. In addition, this article shows that variability in the efficiency may be due to climatology since both in this study and in others existing in the Mediterranean region, important variations in pavement behavior have been detected as a function of initial soil saturation. This indicates that in this climatic region, characterized by a very heterogeneous rainfall regime, soil saturation is even more important than clogging in the first years of operation for efficiencies. This information will help to manage and maintain these pavements in these regions.

## Figures and Tables

**Table 1:** Curve Number (CN) and area of the permeable pavements.

Characterization Data	P1; Block	P2; Concrete Grid	P3; Plastic Grid
Surface (m^2^)	310	310	193
Curve Number (CN)	70	62	60

**Table 2:** Extreme rain events description.

Rain Events Description	Rainfall Depth (mm)	Intensity (mm/min)	Duration (h)
Maximum value	137.20	1.44	35.50
Minimum value	0.17	0.03	0.25

**Table 3:** Statistical rain events description.

Rainfall Depth (mm) Δt = 5 (min)	
Average	0.3714
Median	0.2000
Standard deviation	0.4191
Variance	0.1700

**Table 4:** Definition of efficiency parameters.

Efficiency Parameter (%)	Definition	Equation
Time efficiency (Ɛ_T_)	Increase in water residence time generated by the permeable pavement compared to a conventional pavement	$\varepsilon t=\left(\frac{Tsuds-Tpo}{Tsuds}\right)$
Flow efficiency (Ɛ_F_)	Reduction in peak flow to the sewer system generated by the permeable pavement compared to a conventional pavement	$\varepsilon f=\left(\frac{Fpo-Fsuds}{Fpo}\right)$
Volume ^1^ efficiency (Ɛ_V_)	Reduction in volume flowing to the sewer system by the permeable pavement compared to a conventional pavement	$\varepsilon v=\left(\frac{Vpo-Vsuds}{Vpo}\right)$

^1^ Volume = Drainage volume + Runoff volume.

**Table 5:** Efficiency variability.

Pavements	Medians Variability (%)			Dispersion Data (%)		
	Ɛ_F_	Ɛ_V_	Ɛ_T_	Ɛ_F_	Ɛ_V_	Ɛ_T_
P1	23	56	13	20	60	40
P2	2	26	28	12	40	40
P3	10	98	32	35	60	35

## References

[1] Pistocchi A., Calzolari C., Malucelli F., Ungaro F. (2015). Soil sealing and flood risks in the plains of Emilia-Romagna, Italy. J. Hydrol. Reg. Stud..

[2] European Commission Guidelines on Best Practice to Limit, Mitigate or Compensate Soil Sealing; Commission Staff Working Document. http://ec.europa.eu/environment/soil/pdf/soil_sealing_guidelines_en.pdf.

[3] Fini A., Frangi P., Mori J., Donzelli D., Ferrini F. (2017). Nature based solutions to mitigate soil sealing in urban areas: Results from a 4-year study comparing permeable, porous, and impermeable pavements. Environ. Res..

[4] Scalenghe R., Marsan F.A. (2009). The anthropogenic sealing of soils in urban areas. Landsc. Urban Plan..

[5] Tóth G., Montanarella L., Rusco E. (2008). Threats to Soil Quality in Europe.

[6] Arnfield A.J. (2003). Two decades of urban climate research: A review of turbulence, exchanges of energy and water, and the urban heat island. Int. J. Clim..

[7] Burns M.J., Fletcher T.D., Walsh C.J., Ladson A.R., Hatt B.E. (2012). Hydrologic shortcomings of conventional urban stormwater management and opportunities for reform. Landsc. Urban Plan..

[8] Burton G.A., Pitt R. (2001). Stormwater Effects Handbook: A Toolbox for Watershed Managers, Scientists, and Engineers.

[9] Shuster W.D., Bonta J., Thurston H., Warnemuende E., Smith D.R. (2005). Impacts of impervious surface on watershed hydrology: A review. Urban Water J..

[10] Semadeni-Davies A., Hernebring C., Svensson G., Gustafsson L.-G. (2008). The impacts of climate change and urbanisation on drainage in Helsingborg, Sweden: Combined sewer system. J. Hydrol..

[11] Zhou Q. (2014). A Review of Sustainable Urban Drainage Systems Considering the Climate Change and Urbanization Impacts. Water.

[12] Palla A., Gnecco I. (2015). Hydrologic modeling of Low Impact Development systems at the urban catchment scale. J. Hydrol..

[13] Woods-Ballard B., Wilson S., Udale-Clarke H., Illman S., Scott T., Ashley R., Kellagher R. (2015). The SuDS Manual.

[14] Ahiablame L.M., Engel B.A., Chaubey I. (2012). Effectiveness of Low Impact Development Practices: Literature Review and Suggestions for Future Research. Water Air Soil Poll..

[15] Bressy A., Gromaire M., Lorgeoux C., Saad M., Leroy F., Chebbo G. (2014). Efficiency of source control systems for reducing runoff pollutant loads: Feedback on experimental catchments within Paris conurbation. Water Res..

[16] Charlesworth S., Perales-Momparler S., Lashford C., Warwick F. (2013). The sustainable management of surface water at the building scale: Preliminary results of case studies in the UK and Spain. J. Water Supply Res. Technol..

[17] Norton B.A., Coutts A.M., Livesley S.J., Harris R.J., Hunter A.M., Williams N.S. (2015). Planning for cooler cities: A framework to prioritise green infrastructure to mitigate high temperatures in urban landscapes. Landsc. Urban Plan..

[18] Rodríguez M.I., Cuevas M.M., Huertas F., Martinez G., Moreno B. (2015). Indicators to evaluate water sensitive urban design in urban planning. WIT Transact. Built Environ..

[19] Barbosa A., Fernandes J., David L. (2012). Key issues for sustainable urban stormwater management. Water Res..

[20] Fletcher T.D., Shuster W., Hunt W.F., Ashley R., Butler D., Arthur S., Trowsdale S., Barraud S., Semadeni-Davies A., Bertrand-Krajewski J.-L. (2014). SUDS, LID, BMPs, WSUD and more—The evolution and application of terminology surrounding urban drainage. Urban Water J..

[21] Cettner A., Ashley R., Viklander M., Nilsson K. (2013). Stormwater management and urban planning: Lessons from 40 years of innovation. J. Environ. Plan. Manag..

[22] Lundy L., Wade R. (2011). Integrating sciences to sustain urban ecosystem services. Prog. Phys. Geogr. Earth Environ..

[23] Newman R., Ashley R., Cettner A., Viklander M. The role of context in framing discourses in the transition from piped to sustainable stormwater systems. Proceedings of the NOVATECH 2013.

[24] Roy A.H., Wenger S.J., Fletcher T.D., Walsh C.J., Ladson A.R., Shuster W.D., Thurston H.W., Brown R.R. (2008). Impediments and Solutions to Sustainable, Watershed-Scale Urban Stormwater Management: Lessons from Australia and the United States. Environ. Manag..

[25] Brown R.A., Borst M. (2014). Quantifying evaporation in a permeable pavement system. Hydrol. Process..

[26] Hunt D.V.L., Rogers C. (2005). Barriers to sustainable infrastructure in urban regeneration. Proc. Inst. Civ. Eng. Eng. Sustain..

[27] Perales-Momparler S., Andrés-Domenech I., Andreu J., Escuder-Bueno I. (2015). A regenerative urban stormwater management methodology: The journey of a Mediterranean city. J. Clean. Prod..

[28] McLaughlin A.-M., Charlesworth S., Coupe S., de Miguel E. Resilience and sustainable drainage: End-of-life. Proceedings of the NOVATECH 2016.

[29] Sañudo-Fontaneda L., Andrés-Valeri V.C., Costales-Campa C., Cabezon-Jimenez I., Cadenas-Fernandez F. (2018). The Long-Term Hydrological Performance of Permeable Pavement Systems in Northern Spain: An Approach to the “End-of-Life” Concept. Water.

[30] Braswell A.S., Winston R.J., Hunt W.F. (2018). Hydrologic and water quality performance of permeable pavement with internal water storage over a clay soil in Durham, North Carolina. J. Environ. Manag..

[31] Liu Y., Li T., Yu L. (2020). Urban heat island mitigation and hydrology performance of innovative permeable pavement: A pilot-scale study. J. Clean. Prod..

[32] Selbig W.R., Buer N.H., Danz M.E. (2019). Stormwater-quality performance of lined permeable pavement systems. J. Environ. Manag..

[33] Lucke T., Beecham S. (2011). Field investigation of clogging in a permeable pavement system. Build. Res. Inf..

[34] Nichols P.D., Lucke T. (2017). A Detailed Analysis of Sediment Particle Sizes and Clogging in Permeable Pavements. CLEAN Soil Air Water.

[35] Kumar K., Kozak J., Hundal L., Cox A., Zhang H., Granato T. (2016). In-situ infiltration performance of different permeable pavements in a employee used parking lot—A four-year study. J. Environ. Manag..

[36] Boogaard F., Lucke T., Beecham S. (2013). Effect of Age of Permeable Pavements on Their Infiltration Function. CLEAN Soil Air Water.

[37] Rodríguez-Rojas M., Huertas-Fernández F., Moreno B., Martínez G., Grindlay A. (2018). A study of the application of permeable pavements as a sustainable technique for the mitigation of soil sealing in cities: A case study in the south of Spain. J. Environ. Manag..

[38] USDA (1986). Urban Hydrology for Small Watersheds.

[39] AEMET, Agencia Estatal de Meteorología de España Registros Climáticos. http://www.aemet.es/es/idi/clima/registros_climaticos.

[40] (1999). United States environmental protection agency. Filtr. Sep..

[41] Rossman L.A. (2010). Storm Water Management Model User’s Manual Version 5.0.

[42] National Institute of Standards and Technology Specifications, Tolerances, and Other Technical Requirements for Weighing and Measuring Devices. https://www.nist.gov/system/files/documents/2017/04/28/hb44-15-web-final.pdf.

[43] Molnar P. (2011). Calibration. Watershed Modelling.

[44] Rey S.J., Anselin L. (2009). PySAL: A Python Library of Spatial Analytical Methods. Handbook of Applied Spatial Analysis.

[45] Fernández-Barrera A.H., Castro-Fresno D., Rodriguez-Hernandez J., Calzada-Perez M. (2008). Infiltration Capacity Assessment of Urban Pavements Using the LCS Permeameter and the CP Infiltrometer. J. Irrig. Drain. Eng..

[46] Li H., Kayhanian M., Harvey J. (2013). Comparative field permeability measurement of permeable pavements using ASTM C1701 and NCAT permeameter methods. J. Environ. Manag..

[47] Turco M., Kodešová R., Brunetti G., Nikodem A., Fér M., Piro P. (2017). Unsaturated hydraulic behaviour of a permeable pavement: Laboratory investigation and numerical analysis by using the HYDRUS-2D model. J. Hydrol..

[48] Huang J., He J., Valeo C., Chu A. (2016). Temporal evolution modeling of hydraulic and water quality performance of permeable pavements. J. Hydrol..

[49] Kamali M., Delkash M., Tajrishy M. (2017). Evaluation of permeable pavement responses to urban surface runoff. J. Environ. Manag..

[50] Brunetti G., Šimůnek J., Turco M., Piro P. (2017). On the use of surrogate-based modeling for the numerical analysis of Low Impact Development techniques. J. Hydrol..

